# Bispecific antibodies and their applications

**DOI:** 10.1186/s13045-015-0227-0

**Published:** 2015-12-21

**Authors:** Gaowei Fan, Zujian Wang, Mingju Hao, Jinming Li

**Affiliations:** National Center for Clinical Laboratories, Beijing Hospital, No 1 Dahua Road, Dongdan, Beijing, 100730 China; Graduate School, Peking Union Medical College, Chinese Academy of Medical Sciences, Beijing, 100730 China; Shunyi District Maternal and Child Health Hospital of Beijing City, Beijing, 101300 China

**Keywords:** Bispecific antibody, BiTE, Cancer, Formats, Catumaxomab, Diagnosis

## Abstract

Bispecific antibodies (BsAbs) recognize two different epitopes. This dual specificity opens up a wide range of applications, including redirecting T cells to tumor cells, blocking two different signaling pathways simultaneously, dual targeting of different disease mediators, and delivering payloads to targeted sites. The approval of catumaxomab (anti-EpCAM and anti-CD3) and blinatumomab (anti-CD19 and anti-CD3) has become a major milestone in the development of bsAbs. Currently, more than 60 different bsAb formats exist, some of them making their way into the clinical pipeline. This review summarizes diverse formats of bsAbs and their clinical applications and sheds light on strategies to optimize the design of bsAbs.

## Background

Currently, 44 monoclonal antibody (mAb)-based products are marketed, which generated approximately $75 billion USD in total worldwide sales in 2013 [1]. Therapeutic antibodies have become a mainstay of therapeutic options for patients with cancer and autoimmune, inflammatory, and various other diseases [2, 3]. However, mAbs have several limitations. Patients receiving mAb therapy may develop drug resistance or fail to respond to treatment [4]. Cancer and other diseases are multifactorial, with many signaling pathways implicated in pathogenesis. Single-target immunotherapy does not seem to destroy cancer cells sufficiently. Bispecific antibodies (BsAbs) have been posited as potential cancer therapeutic agents for decades but have only recently begun to bear fruit. BsAbs show several advantages [5]: (1) bsAbs can redirect specific immune cells to the tumor cells to enhance tumor killing, (2) bsAbs will enable the simultaneous blocking of two different mediators/pathways that exert unique or overlapping functions in pathogenesis, and (3) bsAbs can potentially increase binding specificity by interacting with two different cell-surface antigens instead of one.

The development of bsAbs has long been hampered by manufacturing problems such as product instability, low expression yields, and immunogenicity [6]. Newer formats of bsAbs that are more stable, easier to produce, and less immunogenic have been made available. Currently, over 30 bsAbs are in clinical development, with two of the front-runners approved for the market (Table 1).

**Table 1:** Bispecific antibodies in clinical trials. Information from ClinicalTrials.gov (https://clinicaltrials.gov)

BsAb	Sponsor	Formats	Targets	Biological function	Clinical trial × identifier	Diseases
Catumaxomab	Neovii Biotech	Triomab	EpCAM × CD3	T cell recruitment, Fc-mediated effector function	Approved in EU	EpCAM-positive tumor, malignant ascites
	AGO Study Group				Completed phase IIa, NCT00189345	Platinum refractory epithelial ovarian cancer
	AIO-Studien-gGmbH				Phase II, NCT01504256	Gastric adenocarcinomas
	Grupo Español de Investigación en Cáncer de Ovario				Phase II, NCT01246440	Ovarian cancer
	Gustave Roussy				Phase IINCT01784900	Gastric peritoneal carcinomatosis
Ertumaxomab	Krankenhaus Nordwest	Triomab	HER2 × CD3	T cell recruitment, Fc-mediated effector function	Phase I/II, NCT01569412	Her2/Neu-positive advanced solid tumors
FBTA05	Technische Universität München	TrioMab	CD20 × CD3	T cell recruitment	Phase I/II,NCT01138579	Leukemia
Blinatumomab	Amgen Research (Munich) GmbH	BiTE	CD3 × CD19	T cell recruitment	Approved in USA	ALL
	Amgen Research (Munich) GmbH				Phase I, NCT00274742	Relapsed NHL
	Amgen Research (Munich) GmbH				Phase II, NCT01207388	B cell ALL
	Amgen Research (Munich) GmbH				Phase II, NCT01209286	Relapsed/refractory ALL
	National Cancer Institute				Phase I, NCT02568553	NHL
	National Cancer Institute				Phase II, NCT02143414	Adult B-ALL with t(9;22)(q34;q11.2);BCR-ABL1; untreated adult ALL
	National Cancer Institute				Phase III, NCT02003222	BCR-ABL-negative B lineage ALL
Solitomab (MT110, AMG 110)	Amgen Research (Munich) GmbH	BiTE	CD3 × EpCAM	T cell recruitment	Completed phase I, NCT00635596	Solid tumors
AMG 330	Amgen	BiTE	CD33 × CD3	T cell recruitment	Phase I, NCT02520427	Relapsed/refractory AML
MT112 (BAY2010112)	Bayer	BiTE	PSMA × CD3	T cell recruitment	Phase I, NCT01723475	Prostatic neoplasms
MT111 (MEDI-565)	MedImmune LLC	BiTE	CEA × CD3	T cell recruitment	Completed phase I, NCT01284231	Gastrointestinal adenocarcinomas
BAY2010112	Bayer	BiTE	CD3 × PSMA	T cell recruitment	Phase I, NCT01723475	Prostatic neoplasms
MEDI-565	MedImmune LLC	BiTE	CEA × CD3	T cell recruitment	Completed phase I, NCT01284231	Gastrointestinal adenocarcinomas
MDX447	Dartmouth-Hitchcock Medical Center	2 (Fab’) was crosslinked	CD64 × EGFR	Active monocytes to kill tumor	Completed phase I, NCT00005813	Brain and central nervous system rumors
TF2	Garden State Cancer Center at the Center for Molecular Medicine and Immunology	Dock and lock	CEA × HSG	Enzyme-linked immunosorbent assay	Phase I, NCT00895323	Colorectal cancer
	Centre René Gauducheau			Radioimmunotherapy	Phase I/II, NCT01221675	Small cell lung cancer
	Nantes University Hospital			Immuno-PET	Phase I/II, NCT01730638	Recurrences of medullary thyroid carcinoma
	Nantes University Hospital			Immuno-PET	Phase I/II, NCT01730612	HER2 negative breast carcinoma expressing CEA
	Radboud University			Radioimmunotherapy	Completed phase I, NCT00860860	Colorectal neoplasms
rM28	University Hospital Tuebingen	Tandem scFv	CD28 × HMV-MAA	Retargeting autologous lymphocytes to tumor	Phase I/II, NCT00204594	Malignant melanoma
HER2Bi-aATC	Barbara Ann Karmanos Cancer Institute	T cells preloaded with bsAbs	CD3 × HER2	Activated T cells	Phase I, NCT02470559	Ovarian, fallopian tube, or primary peritoneal cancer
GD2Bi-aATC	Barbara Ann Karmanos Cancer Institute	T cells preloaded with bsAbs	CD3 × GD2	Activated T cells	Phase I/II, NCT02173093	Children and young adults with neuroblastoma and osteosarcoma
	Barbara Ann Karmanos Cancer Institute				Completed phase I, NCT00938626	Multiple myeloma and plasma cell neoplasm
	Barbara Ann Karmanos Cancer Institute				Completed phase 1, NCT00244946	NHL
EGFRBi-aATC	Barbara Ann Karmanos Cancer Institute	T cells preloaded with BsAb	CD3 × EGFR	Autologous activated T cells to EGFR-positive tumor	Phase I/II, NCT02521090	Adult brain glioblastoma; adult gliosarcoma; recurrent brain neoplasm
MGD006	MacroGenics	DART	CD123 × CD3	Retargeting of T cells to tumor	Phase I, NCT02152956	Relapsed/refractory AML
MGD007	MacroGenics	DART	gpA33 × CD3	Retargeting of T cells to tumor	Phase I, NCT02248805	Colorectal carcinoma
MGD010	MacroGenics	DART	CD32B × CD79B		Phase I, NCT02376036	Healthy subjects
Anti-CEAxanti-DTPA	Nantes University Hospital	scFv-IgG	CEA × di-DTPA-131I	Radioimmunotherapy	Complete phase II, NCT00467506	Medullary thyroid carcinoma
DT2219ARL	Masonic Cancer Center	2 scFv linked to diphtheria toxin	CD19 × CD22	Targeting of protein toxin to tumor	Phase I, NCT00889408	Leukemia; lymphoma
	Masonic Cancer Center				Phase I/II, NCT02370160	Relapsed or refractory B lineage leukemia or lymphoma
IMCgp100	Immunocore Ltd	ImmTAC	CD3 × gp100	T cell recruitment	Phase I, NCT01211262	Malignant melanoma
					Phase I, NCT02570308	Uveal melanoma
Indium-labeled IMP-205xm734	Radboud University	Unclear	CEA × in-labeled Peptide	Nuclear imaging	Phase I,NCT0018508	Colorectal cancer
LY3164530	Eli Lilly and Company	OrthoFab-IgG	MET × EGFR	Blockade of 2 receptors	Phase I, NCT02221882	Neoplasms; neoplasm metastasis
OMP-305B83	OncoMed Pharmaceuticals, Inc.	DVD-Ig	DLL4 × VEGF	2-ligand inactivation	Phase I, NCT02298387	Advanced solid tumor malignancies
REGN1979	Regeneron Pharmaceuticals	Unclear	CD20 × CD3	T cell recruitment	Phase I, NCT02290951	CD20+ B cell malignancies
COVA322	Covagen	IgG-fynomer	TNF-α × IL17A	Blockade of two proinflammatory cytokines	Phase I/II, NCT02243787	Plaque psoriasis
RG7802	Hoffmann-La Roche	CrossMab	CEA × CD3	T cell recruitment	Phase I, NCT02324257	Solid cancers
RG7813 (RO6895882)	Hoffmann-La Roche	ScFv-IgG	CEA × IL2	The delivery of cytokines	Phase I, NCT02004106	Advanced and/or metastatic solid CEA+ tumors
RG7221 (RO5520985)	Hoffmann-La Roche	CrossMAb	Ang-2 × VEGF	2-ligand inactivation	Phase II, NCT01688206	Neoplasms
RG7716	Hoffmann-La Roche	CrossMAb	VEGF × Ang-2	2-ligand inactivation	Phase II,NCT02484690	Wet AMD
MM-111	Merrimack Pharmaceuticals	HSA body	HER2 × HER3	Blockade of 2 receptors	Completed phase I, NCT01097460	Breast neoplasms
	Merrimack Pharmaceuticals			Blockade of 2 receptors	Completed phase I, NCT00911898	Her2-amplified solid tumors
MM-141	Merrimack Pharmaceuticals	scFv-IgG	IGF-IR × HER3	Blockade of 2 receptors	Phase I, NCT01733004	Hepatocellular carcinoma
					Phase II, NCT02399137	Pancreatic cancer
MOR209/ES414	Emergent Product Development Seattle LLC	scFv-IgG	PSMA × CD3	T cell recruitment	Phase I, NCT02262910	Prostate cancer
TargomiRs	University of Sydney	Unclear	EGFR × EDV	Delivery of nanoparticles	Phase I, NCT02369198	Recurrent MPM and NSCLC
MSB0010841	Merck KGaA	Nanobody	IL-17A/F	Blockade of 2 proinflammatory cytokines	Phase I, NCT02156466	Psoriasis
ALX-0061	Ablynx	Nanobody	IL-6R × HSA	Blockade of proinflammatory cytokine, binds to HSA to increase half-life	Completed phase I/II, NCT01284569	Rheumatoid arthritis
Ozoralizumab (ATN-103)	Ablynx	Nanobody	TNF × HSA	Blockade of proinflammatory cytokine binds to HSA to increase half-life	Completed phase II, NCT01063803	Rheumatoid arthritis
AFM13	University of Cologne	TandAb	CD30 × CD16A	Active NK cells	Phase II, NCT02321592	Relapsed or refractory Hodgkin lymphoma
AFM11	Affimed GmbH	TandAb	CD30 × CD19	Redirecting of T cells	Phase I, NCT02106091	Relapsed and/or refractory CD19-positive B cell NHL
SAR156597	Sanofi	scFv-IgG	IL4 × IL13	Blockade of proinflammatory cytokines	Completed phase I/II, NCT01529853	Idiopathic pulmonary fibrosis
	Sanofi				Phase II, NCT02345070	Idiopathic pulmonary fibrosis

*bsAb* bispecific antibody, *ALL* lymphoblastic leukemia, *NHL* non-Hodgkin lymphoma, *wet AMD* wet type age-related macular degeneration, *MPM* malignant pleural mesothelioma, *NSCLC* non-small-cell lung cancer

BsAbs are primarily produced by three methods [7]: (1) quadroma technology based on the somatic fusion of two different hybridoma cell lines, (2) chemical conjugation, which involves chemical cross-linkers, and (3) genetic approaches utilizing recombinant DNA technology. These technologies have revolutionized the development of bsAbs, and a large variety of formats have been generated to cater to particular applications, some of which are discussed in this review.

## BsAb formats

BsAbs can be roughly divided into two categories: immunoglobulin G (IgG)-like molecules and non-IgG-like molecules (Fig.1). IgG-like bsAbs retain Fc-mediated effector functions such as antibody-dependent cell-mediated cytotoxicity (ADCC), complement-dependent cytotoxicity (CDC), and antibody-dependent cellular phagocytosis (ADCP) [6]. The Fc region of bsAbs facilitates purification and improves solubility and stability. BsAbs in IgG-like formats usually have longer serum half-lives owing to their larger size and FcRn-mediated recycling [8]. Non-IgG-like bsAbs are smaller in size, leading to enhanced tissue penetration [8].

### IgG-like formats

1. Quadromas
  The quadroma technology relies on the fusion of two distinct hybridomas. The random pairing of Ig heavy and light chains gives rise to bsAbs [9]. In this process, nonfunctional antibodies are also produced. BsAbs produced by quadromas resemble conventional antibodies. Catumaxomab, the first approved bsAb, is produced by a rat/mouse quadroma cell line.
2. Knobs-into-holes
  The production of bsAbs with an Fc region poses some challenges such as the formation of undesirable homodimers and other product-related contaminants including mispaired molecules. The “knobs-into-holes” approach has been adopted to tackle these problems by substituting a large amino acid for a small one in the CH3 domain (the “knob”) of one antibody and vice versa (the “hole”) of the other antibody [10]. In theory, heterodimers of any two different antibodies can pair in a “knob-and-hole” fashion. However, “light chain mispairing” poses another challenge. To circumvent this, several methods have been proposed:
  - Generating bsAbs with common light chains [11]. This strategy, however, limits binding specificities and is not applicable to all bsAbs.
  - Expressing the knob- and the hole-containing half-molecules separately in different bacteria [6]. This method would avoid mispairing of the light chains. However, expression in bacterial cells can also result in the loss of key glycosylation modifications, which may affect antibody effector functions (e.g., antibody-dependent cellular cytotoxicity mediated by carbohydrate-dependent binding to Fcγ receptors) [12].
  - Combining CrossMab and knobs-into-holes strategies to minimize mispairing. In the CrossMab antibody, the CH_1_ domain of the heavy chain is swapped with the constant CL domain of the corresponding light chain to induce the right pairing of the light chains [13]. By combining knobs-into-holes and CrossMab, Roche generated the bsAb A2V CrossMab with dual specificities for Ang-2 and VEGFA [14].
  - Introducing additional mutations into VH-VL and CH1-CL interfaces. These mutations encourage a heavy chain to preferentially pair with a light chain [15]. One drawback, however, is that it requires extensive mutations in the conserved regions of the antibody.
3. Dual-variable domains Ig (DVD-Ig)
  The variable domains of two mAbs are fused in tandem to create a dual-specific IgG-like molecule [16]. Each Fab of the DVD-Ig binds to two targets. In theory, any pair of mAbs can be used to generate a DVD-Ig molecule. The resulting specific antibodies could be further modified to create molecules with variable valencies and specificities. This technology avoids mispairing of different heavy or light chains, and it improves product homogeneity, yield, and stability. Additionally, the Fc domain facilitates efficient purification. However, there is a potential risk that the binding affinity of the inner variable domain may be reduced [17].
4. IgG-single-chain Fv (scFv)
  IgG-scFv is generated by fusing an scFv or a variable single domain to the termini of light or heavy chains. This group of antibodies also includes DVD-Igs.
5. Two-in-one or dual action Fab (DAF) antibodies
  Antigen-binding sites of DAF antibodies are capable of dual antigen recognition [18]. To achieve this, a template antibody binding to a target antigen is first identified. A mutation is then introduced into the antigen-binding site to recognize a second antigen. Further engineering within the antigen-binding site is needed to facilitate high dual affinity. However, two-in-one antibodies are not capable of binding to two different antigens (epitopes) simultaneously.
6. Half-molecule exchange
  Human IgG4 antibodies can exchange half-molecules in the serum, leading to the generation of IgG4 bsAbs by a strategy termed “half-molecule exchange.” After the introduction of point mutations into the IgG1 CH3 domain, IgG1 antibodies can undergo half-molecule exchange under controlled conditions to become bsAbs [19]. This strategy also applies to human IgG2 and IgG3. The foremost advantage of this method is the separate expression of the parental IgGs, thereby increasing the repertoire of parental antibodies to generate different bsAbs. Thus, this method is an elegant way to produce numerous bsAbs in a short period. Moreover, human bsAbs produced by half-molecule exchange are in their natural formats, with low immunogenicity.
7. κλ-bodies
  One heavy chain and two different light chains (one κ and one λ) with different binding specificities can be co-expressed in a single cell. In this way, bsAbs with both κ and λ light chains paring with the same heavy chain can be produced [20] and then purified by highly selective affinity resins. The advantages of κλ-bodies are obvious: firstly, the bsAbs retain the complete human IgG format without modification; secondly, the bsAbs can be easily purified from the mixture of antibodies; thirdly, the bsAbs can be produced at an industrial scale; and finally, the purification platform can be applied to any κλ-body, thus facilitating parallel development of different bsAbs [20].

### Non-IgG-like formats

1. scFv-based bsAbs
  ScFv comprising only the VL and VH is the basic element for antigen binding. ScFvs can become dimers, trimers, or tetramers depending on linker length, antibody sequence, and external factors [21]. Compared to normal IgG molecules, scFvs exhibit high tumor specificity and tissue penetration; thus, scFv-based bsAbs are favored and have several possible clinical applications.
  - Tandem scFvs
    Two scFvs are connected by a flexible peptide linker such as glycine-serine repeat motifs in a tandem orientation [7]. The short linker prevents intra-chain but not inter-chain pairing of the VH and the VL domains. The long flexible linker permits antigen-binding sites to rotate freely. The famous bispecific T cell engager (BiTE) technology is based on this format.
  - Diabody format
    In the diabody format, the variable domains of two different antibodies are connected by two linkers. The VH of the first antibody is linked to the VL of the second antibody, and the VL of the first antibody is linked to the VH of the second antibody. The two linkers increase the stability of the diabody. However, there are trade-offs as the two linkers restrict the mobility of the antigen-binding sites, thus limiting antigen recognizing.
  - Single-chain diabodies
    The diabody format can be converted into a single-chain diabody by adding an additional connection linker between the chains.
  - Tandem diabodies (TandAbs)
    When two pairs of VL and VH domains are connected in a single polypeptide chain, a tetravalent tandAb is formed.
  - Dual-affinity retargeting molecules (DARTs)
    DARTs are created by the association of the VH of a first variable region linked to the VL on a second chain, and the VH of the second variable region linked to the VL on the first chain in a VL_A_ − VH_B_ + VL_B_ − VH_A_ configuration [22]. An inter-chain disulfide bond is introduced to stabilize the diabody [22]. The small size of DARTs makes them prone to elimination. To avoid this, MacroGenics fused an Fc fragment to the DARTs to prolong their serum retention time [23].
2. Nanobodies
  Nanobodies are the smallest naturally occurring antibodies and consist only of a heavy chain (15 kDa) [24]. A nanobody can bind to the corresponding antigen in the absence of a light chain. Nanobodies with different binding specificities obtained from llamas and camels have been connected with short linkers to create bsAbs [25].
3. Dock-and-lock (DNL) method
  In this method, antibody fragments are fused to heterodimerizing proteins such as cAMP-dependent protein kinase A (PKA) and A kinase-anchoring protein (AKAP). When the proteins heterodimerize, bispecific molecules are generated [26].
4. Other bispecific/multispecific molecules
  ScFvs can also be connected to other molecules such as cytokine TNF-α (TNF-α naturally exists in trimeric form) and the trimerization domain of collagen XV or XVIII as well as zipper dimerization domains (Fos or Jun) to generate multivalent molecules [6].

### Half-life extension strategies

ScFv-based bsAbs have many advantages including ease of manufacturing and enhanced tissue penetration. Additionally, they can bind to epitopes that may be sterically inaccessible to antibodies in complete IgG format. Further, they are less immunogenic owing to the lack of an Fc region, thus avoiding uptake by FcR. However, scFv-based bsAbs suffer from several drawbacks due to their short half-lives, such as rapid blood clearance, fast off-rates, and poor retention times in targeted sites (e.g., tumors). In clinical applications, a short serum half-life increases the number of applications and the doses of therapeutic agents [27]. Thus, extension of the serum half-life can be both economically and therapeutically beneficial. Several strategies are available to extend serum half-lives of bsAbs, such as:

1. Polyethylene glycol (PEG)ylation
  Attaching highly flexible, hydrophilic molecules, such as PEG, will increase the hydrodynamic volume of the bsAbs, thus improving their serum half-lives. However, the number and size of attached PEG chains can lead to partial inactivation or decreased binding affinity of the antibodies [28]. Conjugating a single PEG chain using a site-directed approach appears to be an ideal strategy [27].
2. Fusion with human serum albumin (HSA) or an albumin-binding moiety
  Fusion of scFvs to HSA or an albumin-binding moiety can prolong their serum half-lives. Additionally, HSA interacts with FcRn without altering the antigen-binding affinity. This strategy has been widely adopted by antibody engineers. Albumin fusion/binding does not only increase the molecule size, but it also promotes recycling of the bsAbs, extending their half-lives. Albumin taken up by cells will first bind to the FcRn of the early endosome, thus escaping degradation. Then, albumin is redirected to the plasma membrane and released back into the blood plasma [27]. Merrimack Pharmaceuticals developed MM-111 bsAb by binding scFvs to HSA. Ozoralizumab (ATN-103, Ablynx) is a trivalent bispecific nanobody derived from a camelid heavy chain with a molecular weight of 38 kDa. In theory, molecules of such small size are easily eliminated by the kidneys. To address this problem, ozoralizumab has been designed to bind to HSA while retaining its binding specificity for TNF-α [29].
3. Fc fragment fusion
  Some antibody engineers fuse an Fc fragment to scFv-based bsAbs. The Fc fragment not only improves the molecule size, but it also promotes recycling through FcRn. MacroGenics developed Fc-bearing DART MGD007 for patients with colon cancer.
4. Multimerization
  Multimerization is another strategy to optimize the half-life by modulating the sizes and the binding values of a bsAb.

## Clinical applications of bsAbs

### BsAbs redirecting immune effector cells to the proximity of tumor cells

Cytotoxic T lymphocytes play an important role in the immune response against cancer [30]. However, tumor-specific T cell responses are limited by immune escape mechanisms utilized by tumor cells during immunoediting. Progress in immunotherapy over the past years has allowed overcoming this challenge. One strategy to harness the immune cells is to take advantage of bsAbs to kill tumor cells. Several bsAbs in clinical development are designed to redirect T cells to tumor cells [31]. This process is accompanied by the formation of a transient cytolytic synapse between the T cell and the targeted tumor cell. The subsequent activation and proliferation of T cells leads to tumor cells lysis [32]. Besides T cells, other immune cells such as macrophages, monocytes, granulocytes, and natural killer (NK) cells also exert tumor-killing effects. A number of bsAbs including Triomab, BiTE, DART, and FynomAb provide new treatment options for patients.

1. Triomab antibodies redirecting T cells to tumor cells
  Triomab antibodies are produced with high yield and purity by mouse-rat hybridomas. The desired bsAbs are mixed with by-products such as monospecific antibodies, and L-chain mispairing further complicates purification. Given the fact that light (heavy) chains from rat and mouse associate preferentially, L/H-chain mispairing can be reduced to 4–10 % [33]. The bsAbs are easily purified by protein A [5]. Triomab antibodies are trifunctional, with one arm binding to tumor-associated antigen, the second arm binding to CD3 on T cells, and the chimeric Fc region preferentially recognizing type I (CD64), IIα (CD32a), III Fcγ (CD16) receptor (FcγR) on accessory cells such as macrophages, dendritic cells, and NK cells. T cells are then activated, accompanied by the release of T cell cytokines such as TNF-α and IFN-γ. Additionally, FcγR-positive accessory cells are redirected to tumor cells with the release of high levels of proinflammatory cytokines such as IL-6, IL-12, GM-CSF, and DC-CK1 [34]. Tumor cells are killed through T cell-mediated lysis and ADCC as well as through phagocytosis by activated accessory cells [9] (Fig. 2).

**Fig. 1:**
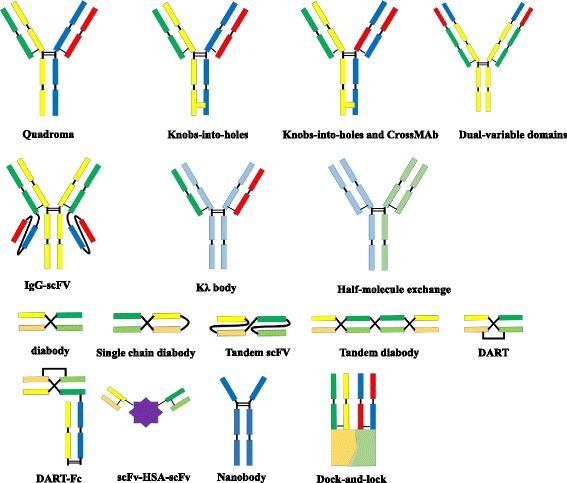
Molecular formats of bispecific antibodies
  Catumaxomab belongs to the Triomab family and was the first bsAb to be approved for cancer treatment. It is produced by co-expression of rat IgG2b and mouse IgG2a in a single host cell. Compared to the BiTE, the IgG-like bsAb has a longer serum half-life. Patients receive intraperitoneal infusion of 10–150 μg four to five times over 9–13 days [8]. Most patients develop a tolerable humoral immune response against catumaxomab. This anti-drug response correlates with a favorable clinical outcome. This protection relies on the presence of the chimeric Fc domain of catumaxomab, which evokes an immunogenic reaction [9]. Potential adverse events suffered by patients include transient fever, nausea, and vomiting. Most adverse events may be attributed to cytokine-release-related symptoms and are reversible [9]. Catumaxomab is approved in the European Union for EpCAM-positive carcinoma patients for whom standard therapy is not feasible. Catumaxomab is currently in clinical trials for application to ovarian cancer, gastrointestinal cancer, non-small cell lung cancer, breast cancer, and peritoneal carcinomatosis [35].
  Other Triomab members include ertumaxomab and FBTA05. Ertumaxomab targets HER2/neu, a validated breast tumor biomarker. By retargeting T cells to HER2/neu-overexpressing cells, ertumaxomab can kill tumor cells with low surface expression of HER2/neu as well [36]. In a phase I study, five out of 15 metastatic breast cancer patients receiving ertumaxomab treatment showed an antitumor response [37]. FBTA05 specifically binds to CD20 on B cells and CD3 on T cells and is in phases I–II trials in patients with CD20-positive B cell malignancies [38].
2. BiTEs redirecting T cells to tumor cells
  The BiTE platform is utilized to develop tandem scFv bsAbs. Blinatumomab binds to CD3 on T cells and CD19-expressing B cell malignancies [39]. It is the first bsAb approved by the US Food and Drug Administration (FDA) for acute B cell lymphoblastic leukemia and has a small size of 55 kDa [40]. Additionally, it is in phases II and III for acute lymphoblastic leukemia (ALL), phase II for diffuse large B cell lymphoma (DLBCL), and phase I for non-Hodgkin lymphoma (NHL). Blinatumomab shows potent tumor-killing capacity by redirecting T cells to tumor cells (Fig. 3). The resulting influx of various granzyme proteases provide essential components for the cytolytic synapse formed between the T cells and target cells [41]. Meanwhile, T cells begin to proliferate and release cytokines such as TNF-α, IFN-γ, IL-6, IL-2, IL-4, and IL-10 [42]. The BiTE molecule is quite potent in redirecting T cells to CD19 positive lymphoma cells at very low concentrations of 10 to 100 pg/ml. Doses of >15 μg/m^2^/day lead to the depletion of tumor cells in vivo. Blinatumomab has a serum half-life of less than 2 h. Patients receive the BiTE infusion via an implanted port system and have to stay under observation for 3–7 days. The treatment resulted in an impressive 43 % complete response rate and a median overall survival of 6.1 months in a phase II trial in patients with high-burden relapsed or refractory B cell ALL (B-ALL) [23]. Blinatumomab has some disadvantages, as some patients suffer from neurotoxicity and show symptoms of cytokine-release syndrome [23]. Due to its small size, blinatumomab can be easily eliminated by the kidneys, and patients need to change the infusion bag every 48 h [32]. It should be noted that some patients receiving blinatumomab develop drug resistance [43]. The underlying mechanisms include, but are not limited to, loss of CD19, extramedullary relapse, and upregulation of programmed death-ligand 1 on tumor cells [43].

**Fig. 2:**
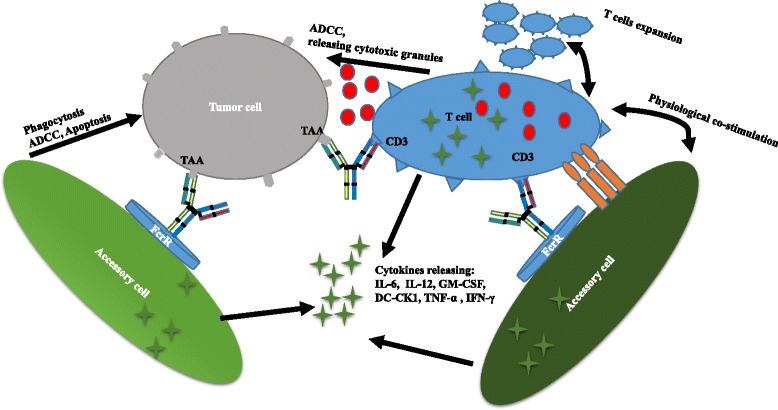
Triomab® antibodies redirect T cells and other accessory cells to a tumor cell

**Fig. 3:**
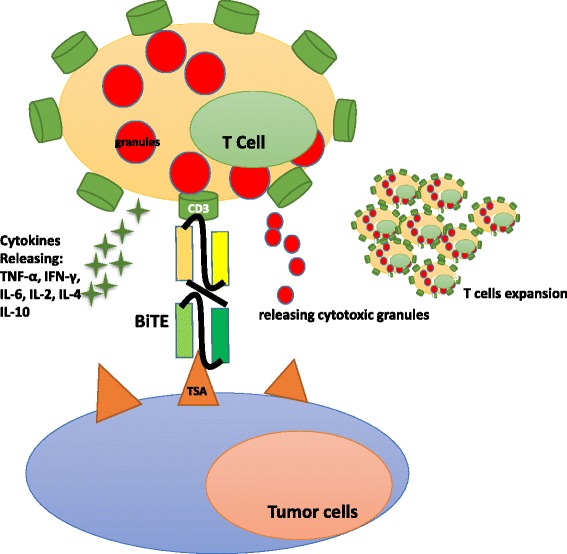
BiTE® antibodies redirect T cells to a tumor cell
  Besides blinatumomab, some other BiTEs such as solitomab (anti-CD3 × EpCAM, completed phase I for solid tumors), MEDI-565 (anti-CD3 × CEA, completed phase I for gastrointestinal adenocarcinoma), and BAY2010112 (anti-CD3 × PSMA, phase I for prostate cancer) are also under investigation [44].
3. DARTs retargeting T cells to tumor cells
  The DART technology is used to produce bsAbs with increased stability and reduced immunogenicity owning to minimal linker size. Unlike BiTEs, the covalent linkage between the two chains of DARTs limits the freedom of the antigen-binding sites. Therefore, DARTs are structurally compact and can form stable contacts between target and effector cells. Moore et al. demonstrated the CD19 × CD3 DART to be more potent than the BiTE molecule in redirected killing of B cell lymphoma [22]. This may be explained by the fact that the binding affinity of the DART format for CD3 is higher and the dissociation rate constant for CD19 is lower [45]. However, with only one study conducting a side-by-side comparison available, it is hard to conclude which format is better. More clinical trials are needed to assess the superiority between the DART and BiTE formats [45]. However, Moore et al. demonstrated that DART could be an alternate T cell activator.
  Both mammalian and prokaryotic systems can be used for antibody expression. To date, more than 70 DART products have been generated [5]. Various mechanisms including activation of tumor-killing effector cells, targeting of receptors or cytokines, and binding to pathogenic epitopes are involved in DARTs function. Two products are currently in clinical trials. MGD006 is designed to treat acute myeloid leukemia (AML) by redirecting T cells to kill leukemic cells [46]. It has two specific targeting arms: one for CD123 on leukemic stem cells and the other one for CD3 on T cells. Currently, MGD006 is in phase I in patients with AML.
  Unlike MGD006, MGD007 belongs to the Fc-bearing DARTs. Evidently, the fusion of the Fc fragment avoids easy clearance and prolongs the serum retention time by FcRn-dependent recycling. MGD007 redirects T cells to gpA33-positive colon cancers and mediates potent lysis of gaA33-positive cells [47]. It is now in phase I trial in patients with colorectal cancer.
4. TandAbs redirecting immune cells to kill tumor
  TandAbs comprise four binding sites without an Fc fragment. Larger than BiTEs, they have a molecular weight of about 114 kDa [48]. Therefore, they have longer serum half-lives than BiTEs and diabodies. TandAbs can be produced in bacterial and mammalian cells. A major difference with BiTEs is that TandAbs exhibit bivalent binding activity for each specificity—an important means to increase target-binding affinity. They are effective at retargeting immune cells to tumor cells and inducing cell lysis.
  AFM13 is a tetravalent bsAb with a murine anti-CD30 domain. It specifically targets CD16A on NK cells and macrophages [49]. CD16A is an activating receptor involved in tumor-cell killing. In Hodgkin’s patients, CD30 is highly expressed by Hodgkin and Reedsternberg cells. AFM13 recruits and activates NK cells to induce lysis of CD30-positive tumor cells. Dose-limiting toxicity such as hemolytic anemia may be seen in patients receiving AFM13. Further, an anti-drug response has been observed in some patients [49]. Currently, AFM13 is in clinical phase II in patients with Hodgkin’s disease. Unlike AFM13, AFM11 redirects T cells to CD19 positive lymphomas. AFM11 is in clinical phase I trial in patients with non-Hodgkin’s lymphoma and ALL.

## BsAbs blocking signaling pathways

As a subclass of cell-surface growth factor receptors, receptor tyrosine kinases (RTKs) are critically involved in oncogenesis [50]. Several monospecific RTK-targeting antibodies including herceptin (for metastatic breast cancer), imatinib (for CML and GIST), gefitinib (for NSCLC), and cetuximab (for colorectal cancer) have been approved for cancer therapeutics [50]. However, multiple signaling pathways exerting unique or overlapping functions are involved in pathogenesis. Thus, simultaneously neutralizing two targets with one molecule exhibits unique appeal and offers better treatment potential than mAbs.

1. HSA body bsAbs
  MM-111 is a bsAb with two scFvs fused to modified HSA [51]. It targets the HER2/HER3 signaling pathways simultaneously. HER2 is a validated target for numerous cancers. HER3 signaling is an important mechanism of drug resistance to HER2 inhibitor. Dual targeting of HER2/HER3 can lead to a more effective response. MM-111 alone or with trastuzumab is in clinical trials in patients with HER2-postitive solid tumors [27].
2. ScFv-IgGs
  MM-141 is another bsAb targeting the insulin-like growth factor I receptor (IGF-IR) and HER3. Unlike MM-111, MM-141 is an IgG-like bsAb with two scFvs fused to the constant region of an IgG. Both IGF-IR and HER3 activate the PI3K/AKT/mTOR axis—a mechanism for targeted resistance. MM-141 binds to IGF-IR and HER3, thus blocking the downstream resistance mechanism [52]. MM-141 is currently in phase I study in patients with hepatocellular carcinoma.
3. Two-in-one antibody
  Duligotuzumab is a two-in-one (DAF) phage-derived humanized antibody. It binds to EGFR and HER3, resulting in the inhibition of the downstream signaling pathways of HER-family [53]. Deregulated EGFR- and HER3-dependent signaling is involved in the pathogenesis of human cancers such as head and neck and colorectal cancers. Patients who receive EGFR inhibitor cetuximab treatment often develop anti-EGFR resistance. When used in combination with radiation, duligotuzumab overcomes drug resistance and enhances the effects of radiation. Currently, duligotuzumab is in clinical trials in patients with epithelial tumors and neck squamous cell carcinoma. Duligotuzumab exhibited similar antitumor activity as cetuximab in a phase II study in patients with recurrent or metastatic neck squamous cell carcinoma [54]. It should be noted that patients receiving duligotuzumab are at high risk of adverse effects such as febrile neutropenia, hypokalemia, nausea, and dehydration [55].

## BsAbs targeting tumor angiogenesis

Angiogenesis is a key process in tumor growth and metastasis. Multiple angiogenic factors including endothelial growth factor receptor 2 (VEGFR2), VEGFR3, endothelial growth factor A (VEGFA), angiopoietins, and platelet-derived growth factors (PDGFs) are involved in tumor angiogenesis. Many cancer therapies disrupt angiogenesis by depleting these proteins [56]. Dual targeting of angiogenic factors leads to superior outcomes [57].

RG7221 is a human IgG1-like CrossMab targeting two key angiogenic factors, VEGFA and angiopoietin-2 (Ang-2). In preclinical models, RG7221 strongly inhibited angiogenesis and tumor growth, with superior effect as comparing to single-pathway inhibitors [14]. Currently, RG7221 is in phase II study in patients with colorectal cancer. According to clinical data from the phase I study, patients receiving RG7221 treatment may suffer from hypertension, asthenia, headache and fatigue [14].

RG7716 is a similar CrossMab, which was also designed to block VEGFA and Ang2, and is in phase II study in patients with wet type age-related macular degeneration (wet AMD).

## BsAbs blocking cytokines

Several cytokines have been identified as key mediators of inflammatory and autoimmune diseases [58]. Therefore, blockage of these cytokines has treatment potential. For example, the inhibition of TNF-α exerts profound therapeutic effects on psoriasis, psoriatic arthritis, Crohn’s disease, ulcerative colitis, juvenile arthritis, and many other diseases [59]. Other validated cytokines include IL-6, IL-17, IL-1, IL-12, TGF-β, IL-4, and IL-13. [59–62].

### Nanobodies

Ozoralizumab (ATN-103) is a small trivalent, bispecific nanobody developed by Ablynx with high affinity for TNF-α and HSA. Albumin binding increases its serum half-life [63]. Ozoralizumab completed phase II clinical trial in patients with rheumatoid arthritis (RA), and it showed significant improvement in RA. Ozoralizumab exhibits specific molecular features such as small size, low immunogenicity, and long serum half-life, making it appealing for clinical applications.

Similar nanobodies developed by Ablynx include ALX-0061 against IL-6R/HSA for RA and ALX-0761 against IL-17A/F for inflammatory disease [64, 65].

### SAR156597

SAR156597 is a tetravalent bispecific tandem IgG that simultaneously binds to IL-13 and IL-4. Structurally, SAR156597 is an IgG molecule (anti-IL4 antibody) with its N terminus fused to the variable domain of an anti-IL13 antibody [66]. It has completed phase I/II clinical investigation for idiopathic pulmonary fibrosis [67].

## BsAbs as delivery vehicles

An interesting application of bsAbs is the delivery of payloads such as drugs, radiolabels, and nanoparticles. The payloads are administered once the unbound bispecific molecules are cleared from the bloodstream. Bispecific molecules can be used to enrich payloads in tumor sites [26, 68–70]. This strategy significantly prolongs the serum retention time and improves the tumor/blood ratio.

### DNL

The bispecific TF2 built made by the DNL method is used for tumor imaging and radioimmunotherapy. It specifically binds to CEA and ^99m^T-labeled hapten histamine-succinyl-glycine (HSG). In the preclinical trial, TF2 was first injected, and ^99m^T-labeled HSG was then administered after the clearance of the bsAb from the blood. A high tumor/blood ratio was observed with high tumor uptake of ^99m^T. At present, TF2 is in phase I study in patients with colorectal cancer [71]. Other applications of TF2 include targeting against ^177Lu^ HSG/^111In^HSG and CEA for radioimmunotherapy in patients with colorectal neoplasms and targeting against ^68Ga^HSG and CEA for immuno-positron emission tomography.

## BsAbs in preclinical development

1. BsAbs crossing the blood-brain barrier
  A promising application of bsAbs is to cross the blood-brain barrier (BBB) to target pathogenesis mediators in neurological diseases [72]. The BBB forms a forbidden zone for monospecific antibody therapy. Couch et al. designed a bsAb that binds to transferrin receptor (TfR) and β-site APP-cleaving enzyme 1 (BACE1) to overcome this hurdle [73]. TfR is highly expressed on the surface of brain endothelium. BACE1 is an aspartyl protease that contributes to the pathogenesis of Alzheimer’s disease, and targeting BACE1 has been a long-sought-after strategy for treating Alzheimer’s disease [74]. After binding to TfR, the circulating bsAb is transported into the brain via receptor-mediated transcytosis. The affinity between the bsAb and TfR is weak; therefore, bsAb can be released from the endothelium and enter the brain to target disease mediator BACE1 with the other binding arm. A preclinical study showed that the bsAb could alleviate disease syndromes [74]. Several other bsAbs are being developed to cross the BBB via transferrin receptor-mediated transcytosis [72].
2. BsAbs for diagnostic assays
  BsAb can be used in diagnostic assays. Typically, a bsAb is designed to bind a specific antigen and a detecting moiety such as horseradish peroxidase. Therefore, bsAb can function as a cross-linker, binding an antigen and reporter molecules simultaneously. In an immunoassay, a monospecific capture antibody is immobilized onto a solid surface and binds to the corresponding antigen in serum. BsAb is then added to bind the captured antigen and a reporter molecule. Such bsAb-based immunoassays have been applied in patients infected with tuberculosis, hepatitis B, *Escherichia coli*, *Bordetella pertussis*, SARS, and other infectious diseases [75]. Additionally, bsAbs have been used in other diagnostic applications such as immunohistochemistry and radioimmunodiagnosis [76]. Compared to monospecific antibodies, bsAbs simplify diagnostic assays and reduce false-positive reactions [76, 77]. BsAb-based diagnostics specifically detect bacterial or viral antigens, instead of antibodies, thereby enabling early-stage detection.
3. BsAbs for the treatment of pathogens
  Due to the overusing broad-spectrum antibiotics, many pathogenic strains have become antibiotic resistant, and some have even become resistant to multiple antibiotics and chemotherapeutic agents. The rise of multi-drug resistance poses a major threat to the development of new antibiotic classes. The development of bsAbs offers a strategy to overcome this problem. Recently, a study reported the effectiveness of a new bsAb, BiS4αPa, to treat *Pseudomonas aeruginosa* infections [78]. *P. aeruginosa* remains a significant contributor to hospital-acquired pneumonia and mortality in patients with cystic fibrosis. Antibiotics against single epitopes of *P. aeruginosa* are ineffective due to drug resistance. The bispecific BiS4αPa was designed to bind to Psl, an extracellular polysaccharide that plays an important role in immune evasion and biofilm formation, with one binding arm, and to PcrV, a component involved in the secretion of virulence factors, with the other binding arm. The superior protective activity of BiS4αPa was proven in an animal study, and BiS4αPa is now a clinical candidate for the treatment of *P. aeruginosa* [79].
  Besides BiS4αPa, various other bsAbs have been developed to redirect cytotoxic T lymphocytes to kill HIV [80], protect against HBV infection [81], or promote the clearance of bacteriophages [82].

## Conclusions

BsAbs can not only bridge therapeutics (e.g., T cells, drugs) and targets (e.g., tumor) but also simultaneously block two different pathogenic mediators [83]. In the near future, bsAbs might improve treatment options against cancer, autoimmune diseases, and inflammatory diseases. Two bsAbs have been approved with an impressive treatment profile. The success of bsAbs has captured the attention of pharmaceutical companies, with different companies devising new formats.

Success aside, several critical hurdles remain, as only few formats have successfully moved into clinical trials. Large-scale production and purity are long-term pursuits. The ideal platform should encompass the entire development process from discovery and preclinical studies to clinical material production, to allow rapid discovery of potent lead bsAbs and purification of clinical-grade bsAbs in a short time. Thus, simplifying the structure and production procedure and utilizing a powerful production platform are the keys when designing a bsAb format. The identification of target pairs and bsAbs with potential synergistic effects also poses a big challenge, necessitating a high-throughput approach. Moreover, immunogenicity is a complex issue in drug design and development. In clinical trials, adverse effects are often reported and hamper the success of bsAbs. For example, toxicity of the bispecific 4G7 × H22 leads to the termination of its clinical study (https://clinicaltrials.gov/ct2/show/NCT00014560). Most adverse effects are mainly caused by a “cytokine storm.” With the development of bsAbs, there is hope for the availability and approval of more therapeutic alternatives in future.

## Abbreviations

ADCC: antibody-dependent cell-mediated cytotoxicity
ADCP: antibody-dependent cellular phagocytosis
ALL: lymphoblastic leukemia
BACE1: β-site APP-cleaving enzyme 1
BiTE: bispecific T cell engager
BsAb: bispecific antibody
CDC: complement-dependent cytotoxicity
DARTs: dual-affinity retargeting molecules
DNL: dock-and-lock
IgG: immunoglobulin G
mAb: monoclonal antibody
MPM: malignant pleural mesothelioma
NHL: non-Hodgkin lymphoma
NSCLC: non-small-cell lung cancer
scFv: single-chain Fv
TandAbs: tandem diabodies
wet AMD: wet type age-related macular degeneration
